# Synergistic Effect of F–T Synthetic Wax and Surface-Active Agent Content on the Properties and Foaming Characteristics of Bitumen 50/70

**DOI:** 10.3390/ma14020300

**Published:** 2021-01-08

**Authors:** Mateusz M Iwański

**Affiliations:** Department of Technologies and Durability Concrete, Faculty of Civil Engineering and Architecture, Kielce University of Technology, Al. Tysiąclecia Państwa Polskiego 7, 25-314 Kielce, Poland; matiwanski@tu.kielce.pl

**Keywords:** foamed bitumen, F–T synthetic wax, surface active agent SAA, maximum expansion, half-life

## Abstract

The level of the properties of bituminous mixtures produced with water foamed bitumen relies on the optimum characteristics of the bitumen. One way to achieve the desired characteristics is to modify the bitumen with chemical additives before it is foamed. Bitumen 50/70 treated with a surface-active agent (SAA) at 0.2%, 0.4% and 0.6% and Fischer–Tropsch (F–T) synthetic wax at 1.5%, 2.0%, 2.5% and 3.0% was used in the tests. The effect of the modifiers was investigated by assessing bitumen properties (penetration, softening point, Fraass breaking point and dynamic viscosity at 60 °C, 90 °C and 135 °C) and foam parameters (maximum expansion—ER, half-life—HL). For statistical evaluation of the test results, models of the properties of bitumen 50/70 were developed as a function of the contents of F–T synthetic wax and SAA. It was found that 2.0% F–T wax and 0.6% SAA were optimum contents for achieving the desired standard properties and foam characteristics of the tested binder. The developed models allow determining the composition of the modified binder depending on the required foam characteristics for specific applications in road construction. The recommended composition of the chemical additives used to modify the binder was also established to ensure its optimum properties.

## 1. Introduction

The dynamic development of the road building industry has significantly contributed to the advancement of the existing and newly emerging pavement technologies. The underlying concept is to mix and compact bituminous mixtures at lower temperatures for improved energy efficiency, lower greenhouse footprint and for environmental sustainability. To achieve this aim, various types of chemical additives are required to reduce bitumen viscosity to the level of 0.2 Pas to 2.0 Pas. The additives are used to modify both the standard and rheological properties of the bitumen [1,2,3] to enable the production of mixtures using the Warm Mix Asphalt (WMA) technology. WMA mixing temperature is about 40 °C lower [4,5,6] than in the traditional technology characterized by a temperature range of 160 °C to 180 °C, depending on the binder type used.

Another method of reducing bitumen viscosity is using zeolite [7,8,9] or water [10,11] to foam the bitumen. Zeolite is used in the production of WMA [12,13]. More significant mixing temperature reductions can be achieved using water for bitumen foaming [14,15]. Water-foamed bitumen technology has been developing dynamically since the beginning of the 20th century [16,17]. Three parameters: maximum expansion—ER, the half-life of bitumen foam—HL, and foam index—FI characterize the quality of foamed bitumen [16].

Initially, the technology was primarily used for cold deep recycling of lower pavement layers [16,18,19]. Minimum requirements were defined to ensure the quality of the recycled mixture intended for the base course. The temperature of the recycled material and the ambient temperature play a significant role in the production process, especially during works performed “in situ”. The lower these temperatures are, the higher the characteristics of the foamed bitumen should be [16,17]. A lot of research has been undertaken to provide the most beneficial foam parameters by adding various substances (chemical additives) to the bitumen before foaming [20,21]. Foamed bitumen improves the mechanical properties of the recycled mixture [22,23] and allows wider use of road waste materials, including mineral fillers [24,25]. Attempts to lower the mixing temperature of conventional bituminous mixtures intended for upper pavement layers have revealed a possibility of using water-foamed bitumen in mixtures produced in the Half-Warm Mix Asphalt (HWMA) technology with additives and chemicals for improved physical and mechanical parameters [26,27]. The mixing and compaction temperatures used in the HWMA technology are about 100 °C [28,29]. It has thus been necessary to adapt the requirements for the high-level foam characteristics of the binder so that it can be applied in mixtures produced and compacted at a temperature below 100 °C [30]. Chemical additives have to be used to ensure that the binder achieves increased standard and rheological characteristics. It is crucial to make sure that the additives do not affect compaction at the reduced temperature [31]. Such a situation may occur when Fischer–Tropsch (F–T) synthetic wax is used as an additive to bitumen before foaming. At the mixture temperature below 90 °C, the F–T synthetic wax is in the form of crystals which may hinder the compaction process and thus reduce the pavement service life [32,33]. The continued search for other types of additives has aroused interest in surface-active agents (SAA), which, according to the results of the pilot study, have a very positive effect on the bitumen foam characteristics. Good foam parameters guarantee excellent coating of aggregates by the binder, which in turn provides the mixture with the required resistance to moisture and frost [34]. One limitation of this solution is that the standard and rheological properties of the binder may decrease [20,35,36], and the bituminous mixture will have lower mechanical parameters compared to the mixture that does not contain SAA [37]. The problem may be resolved by adding hydrated lime to the mixture. Hydrated lime is used as a multifunctional additive that ensures adequate resistance of the bituminous mixture to water, frost and permanent deformation [38,39,40].

The search for high values of foam parameters and high levels of standard and rheological properties at the same time should be continued to provide bituminous mixtures with proper durability and resistance to water, frost and rutting [34,41]. Additives such as F–T synthetic wax and SAA added to the bitumen before foaming appear to be the right solution; F–T synthetic wax improves the standard and rheological characteristics of the modified binder within a wide temperature range [42,43] and SAA will play a special role in terms of the mixture compaction temperature [36]. Lower bitumen viscosity will counteract the stiffening effect provided by the binder containing F–T synthetic wax. As a result, the process of mixture compaction will not be affected and during the service life of the pavement, synthetic wax will play an important role in ensuring a high level of resistance to permanent deformation.

## 2. Materials and Methods

### 2.1. Material

Bitumen 50/70 (PKN ORLEN S.A., Płock, Poland) was used in this study. It is commonly used in Central and Eastern Europe for the production of bituminous mixtures for wearing and binding courses. The use of softer bitumen in the mixtures intended for the wearing course is not acceptable since the pavement has to be resistant to permanent deformation [44] on roads under vehicle traffic 2.5 × 10^6^ < ESAL_100 kN_ < 7.3 × 10^6^ (ESAL—equivalent single axle load) [45]. Table 1 compiles the basic properties of the binder.

**Figure 1:**
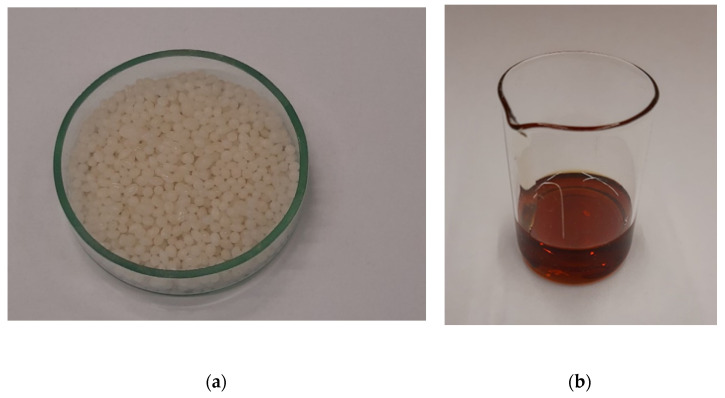
Chemical additives for foaming bitumen 50/70; Fischer–Tropsch (F–T) synthetic wax (**a**), surface-active agent (**b**) (photo by M. M. Iwański).

F–T synthetic wax (Sasol Performance Chemicals, Hamburg, Germany) in the form of very fine granules and a surface-active SAA agent (AkzoNobel, Amsterdam, Netherlands) with a liquid consistency were used as additives, as shown in Figure 1.

Both F–T synthetic wax and SAA significantly reduce bitumen viscosity and thus have a positive effect on the bitumen foam characteristics [3,36]. In addition, they ensure a high level of bitumen adhesion to aggregate, ensuring adequate pavement durability. The properties of synthetic wax F–T and SAA are compiled in Table 2 and Table 3.

Since two different additives were used in the tests, F–T wax in a solid-state and SAA as a liquid, particular attention was paid to the homogeneity of the binder. Proper preparation of the samples was thus especially important, and the order of additive dosing was established. The pilot study demonstrated that proper blending of the added material with the binder is ensured with the addition of SAA followed by the addition of F–T wax. Mixing the binder sample (1000 g in mass) with the additive consisted of first heating the binder to a temperature 100 °C higher than the softening temperature and then mixing it in a blender at that temperature using the speeds of 150 rev/min within 30 s, and then 600 rev/min within 270 s. After that, F–T synthetic wax was added, and the mixing process was repeated. The samples for analysis were prepared as per EN 12594:2014-12 [54].

In the next step, the surfaces of the binder samples were evaluated macroscopically. Where the colour of the binder was not homogeneous, or where different spots were found on the surface of the sample, indicative of an abnormal dissolution of the additives (F–T wax or SAA), the sample was rejected.

The SAA was dosed to the binder at 0.2%, 0.4% and 0.6% by mass of the binder after thorough mixing in the blender [36]. Then F–T synthetic wax was dosed to the binder at 1.5%, 2.0%, 2.5% and 3.0% by mass of the binder [42].

The amount of the WMA additive was determined from the tests conducted earlier:

- SAA was added at 0, 0.2, 0.4 and 0.6% by weight in relations to the binder mass on the basis of the tests described in [36]. The maximum amount of this WMA additive as per the recommendation of the manufacturer is 0.6% and this was the limiting content used in the tests [36];
- F–T synthetic wax was added at 1.5, 2.0, 2.5, and 3.0% by weight in relation to the bitumen mass on the basis on the tests carried out at the Kielce University of Technology and by other authors [29,42,43].

The use of less than 1.5% F–T wax in the bitumen did not significantly affect its foaming characteristics. When more than 3.0% was used, the compaction of the mixture produced in the HWMA technology became problematic. At the compaction temperature, the F–T synthetic wax changed from the liquid to the solid phase, which hindered the process of compacting the mixture.

### 2.2. Experimental Program

The effects of SAA and F–T wax were evaluated in two steps. First, the impact of chemical additives on the standard and rheological properties of the binder before foaming were investigated:

- Penetration at 25 °C (Pen*,* EN 1426:2015-08) [46], which is a measure of the binder consistency;
- Softening point (T_R&B_, EN 1427:2015-08) [47];
- Fraass breaking point (T_Fraass_, EN 12593:2015-08) [48];
- Dynamic viscosity at 60 °C, 90 °C and 135 °C (η, EN 13302:2018) [49].

Rheotest RN 4 rheometer (RHEOTEST Medingen GmbH, Ottendorf-Okrilla, Germany) was used to measure the dynamic viscosity of the binder. Sample preparation and binder tests were performed as per EN 12594:2014-12 [54].

For illustrative purposes, the penetration index values and the temperature plasticity range were also determined.

The penetration index indicates the sensitivity of bitumen to temperature from a measurement of penetration (Pen) at 25 °C and softening point (T_R&B_) according to the formula provided by EN 12591:2009 [55].

(1) $$\mathrm{PI}=\frac{20\cdot T_{R\&B}+500\cdot \log\left(\mathrm{Pen}\right)-1952}{T_{R\&B}+50\cdot \log\left(\mathrm{Pen}\right)-120}$$

The penetration index describes changes in the consistency of the binder against temperature changes. It thus allows evaluating the binder suitability in countries with moderate climate and a high-temperature gradient (high temperatures in summer and low temperatures in winter).

The temperature range of plasticity (PR) of the binder, which depends on its softening point (T_R&B_) and braking point (T_Fraass_), was determined from

(2) $$\mathrm{PR}=T_{R\&B}-T_{\mathrm{Fraass}}\;\left({}^{\circ}C\right)$$

In the second step, the binder foam characteristics were determined:

- Maximum expansion ER [16,17];
- Half-life of the bitumen foam HL [16,17];
- The foam index FI [16].

The physical properties of the bitumen foam were tested at a variable amount of foaming water content (FWC): 1.5%, 2.0%, 2.5%, 3.0%, 3.5% and 4.0%, using the WLB-10S (Wirtgen GmbH, Windhagen, Germany) test stand in accordance with the recommendations set forth in [17].

Then, using the mathematical relationship, the optimum values of ER and HL parameters were calculated as a function of FWC. An important element of the study was to determine the optimum FWC that could guarantee the highest possible expansion index with the longest possible half-life of the foam.

Global assessment of the bitumen foam quality is possible owing to the foam index FI proposed by Jenkins [16]. The FI is measured in seconds and calculated from

(3) $$\mathrm{FI}=\frac{-\mathrm{HL}}{\ln2}\cdot \left(4-{\mathrm{ER}}_{m}-4\ln\left(\frac{4}{{\mathrm{ER}}_{m}}\right)\right)+\left(\frac{1+C}{2C}\right)\cdot {\mathrm{ER}}_{m}\cdot t_{s}\left(s\right)$$

where: C—the correction factor (ER_m_/ER_a_), HL—half-life (s), t_s_—bitumen spraying time (s), ER_m_—the measured expansion ratio (immediately after bitumen foaming), ER_a_—the actual expansion ratio.

The results were subjected to analysis of variance (ANOVA) [56,57] to determine their reliability and analyse the statistical significance of the factors (F–T synthetic wax, SAA).

It was assumed that F–T wax and SAA contents significantly influence the properties of bitumen 50/70 when the p_values_ are lower than the significance level α = 0.05 [56,58].

The experiment was carried out with two factors both at four levels. The goal was to select a mathematical model for establishing the relationship between the output variable “y” and the input variables “xi” using the general formula. Literature review [57,58] indicated the most adequate mathematical model as below

(4) $$y=b_{0}+\overset{n}{\underset{i=1}{\sum }}b_{i}\cdot x_{i}+\overset{n}{\underset{i=j=1}{\sum }}b_{i=j}\cdot x_{i}\cdot x_{j}+\overset{n}{\underset{i=1}{\sum }}b_{\mathrm{ii}}\cdot x_{i}^{2}$$

The response area of this model was a polynomial of the second degree with two factors (x_1_, x_2_) written in the form [57,58]:

(5) $$y=b_{o}+b_{1}\cdot x_{1}+b_{2}\cdot x_{2}+b_{3}\cdot x_{2}\cdot x_{1}+b_{4}\cdot x_{1}^{2}+b_{5}\cdot x_{2}^{2}$$

where:

- y—analysed bitumen parameter
- x_1_—synthetic wax FT (F–T) content in bitumen (%),
- x_2_—surface active agent (SAA) content in bitumen (%),
- b_0_–b_5_—regression coefficients.

**Figure 2:**
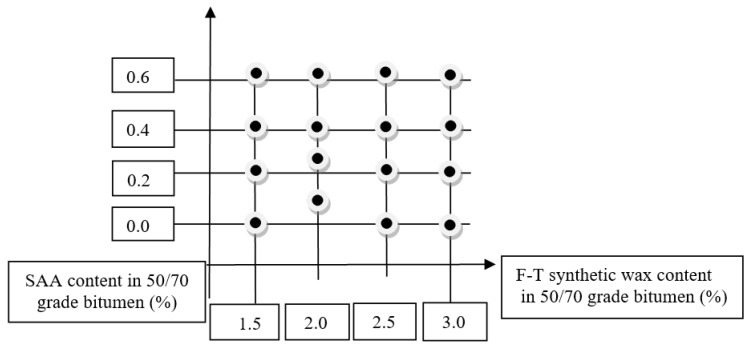
Experimental design.

The research was based on the assumptions of the algorithm of the factorial experimental design [57,58]. The properties of the modified binder, bitumen 50/70, were determined based on the adopted factorial design 4 × 4, as shown in Figure 2.

The results were analysed with the Statistica program (StatSoft Inc., Tulsa, OK, USA) [56] to confirm their reliability and determine significant relationships between the binder parameters and the quantities of F–T wax and SAA.

## 3. Results and Discussion

The evaluation of the impact of F–T wax content and SAA content on the parameters of bitumen 50/70 was presented as models developed based on formula (5). The properties under evaluation comply with the experimental design described in Section 3:

- Penetration at 25 °C (Pen*,* EN 1426:2015-08) [46], which is a measure of binder consistency;
- Softening point (T_R&B_, EN 1427:2015-08) [47];
- Fraass breaking point (T_Fraass_, EN 12593:2015-08) [48];
- Dynamic viscosity at 60 °C, 90 °C and 135 °C (η_60_, η_90_, η_135_, EN 13302:2018) [49];
- Plasticity range PR;
- Penetration index PI;
- aximum expansion ER [16,17];
- Bitumen foam half-life HL [16,17];
- Foam index FI [16].

Parameters of the regression models for investigating the relationship between the parameters under analysis (Pen, T_R&B_, T_Fraass_ η_60_, η_90_, η_135_, PR, PI, ER, HL, FI) and the quantities of F–T synthetic wax and SAA were determined using Statistica [56].

Reliability of the results was ensured by determining each parameter on 9 samples [58].

### 3.1. The Effects of the Synthetic Wax F–T and SAA Content on Basic Bitumen Properties

**Figure 3:**
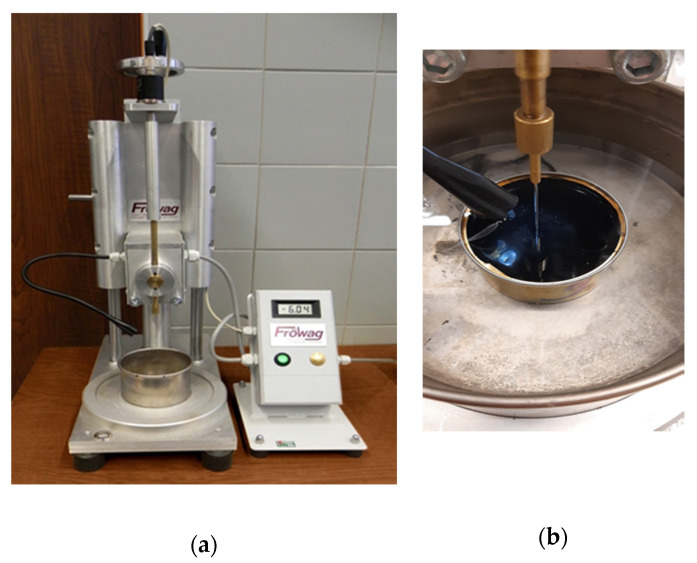
Automatic penetrometer for bitumen testing (**a**), bitumen specimen during testing (**b**) (photo by M. M. Iwański).

Penetration at 25 °C is the basic parameter characterizing the bitumen. The analysis of this parameter allows evaluating its consistency and suitability of the bituminous mixture for a specific pavement layer (wearing course, binding course, base course). The penetration test was performed using the automatic measuring device (FröWag GmbH, Obersulm, Germany) shown in Figure 3.

**Figure 4:**
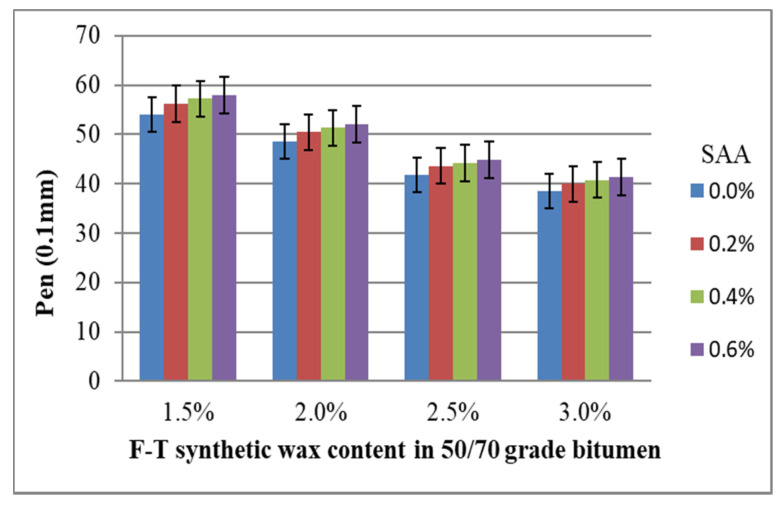
Penetration value of modified bitumen 50/70 versus F–T wax and SAA contents.

The results of penetration at 25 °C tests with respect to F–T synthetic wax and SAA contents were subjected to preliminary statistical analysis. For each test over the entire base of the experiment, the coefficient of variation ranged from 0.8% to 3.1%, which proves the high homogeneity of the results. The average values of the analysed parameter of modified bitumen 50/70 together with the standard deviation are shown in Figure 4.

Analysis of the data in Figure 4 shows that penetration decreases with the increase in the content of the F–T synthetic wax. Bitumen 50/70 stiffens. However, the addition of SAA produces the opposite trend; the penetration value increases. By using both additives, it is thus possible to properly model the penetration value to meet the requirements set for the foamed binder in the bituminous mixture, depending on its intended use. The addition of the F–T synthetic wax diminishes the adverse effect of SAA on the penetration of bitumen 50/70, as reported in [36].

**Figure 5:**
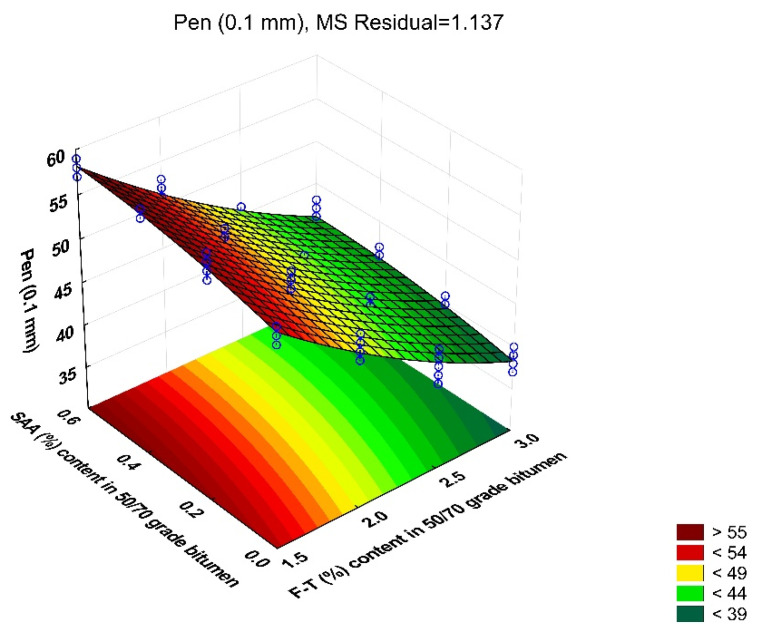
Response surface plot for bitumen 50/70 variability and its model.

Then, a regression model was developed to determine the strength of the relationship between the penetration value of bitumen 50/70 and the contents of F–T synthetic wax and SAA, and the overall effect was evaluated through ANOVA. The values describing the model are summarized in Table 4. A graphical interpretation of the model is shown in Figure 5.

Pen = 80.873 − 21.112 · F–T + 12.529 · SAA − 1.188 · F–T ·SAA + 2.305 · F–T^2^ − 7.465 · SAA^2^ (6)

Analysis of the data in Table 4 allows conclusive indication that the contents of F–T wax and SAA are significant factors impacting bitumen 50/70 penetration because the p_value_ is less than the assumed significance level α = 0.05 (p_value_ < 0.05). The synergistic effect of the F–T wax content and the SAA content on the penetration value is an important observation. The obtained value R^2^ = 0.974 for Pen of the modified bitumen 50/70 indicates a very good fit of the second-degree polynomial variability to the results obtained.

It can be concluded that bitumen 50/70 has the most beneficial penetration levels at the F–T synthetic wax content ranging from 2.5% to 3.0% and at the SAA content from 0.0% to 0.2%. The use of both additives has a better effect on penetration than either of them used alone [20,32,36]. Increasing the amount of SAA increases the penetration of the binder, it becomes softer and, as a consequence, the asphalt concrete made with it may be less resistant to permanent deformation.

**Figure 6:**
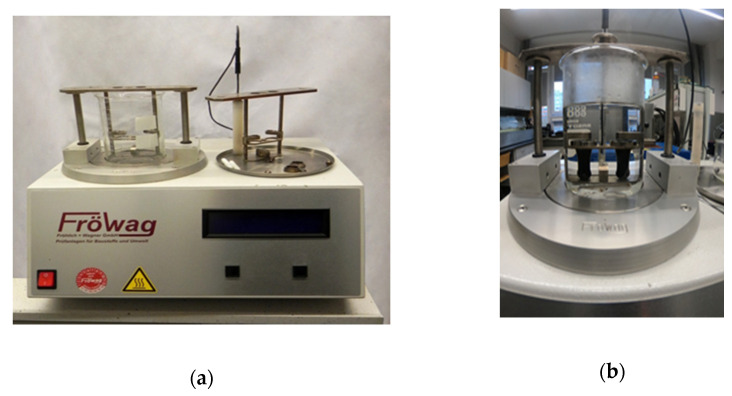
Automatic device for measuring bitumen softening point (**a**), bitumen specimen during testing (**b**) (photo by M. M. Iwański).

Another parameter of analysed in this study is the softening point of the modified bitumen 50/70, determined using the measuring device (FröWag GmbH, Obersulm, Germany) shown in Figure 6.

**Figure 7:**
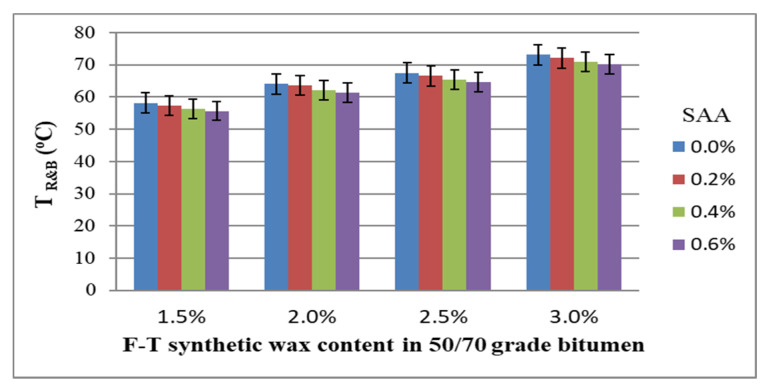
Bitumen 50/70 softening point versus F–T wax and SAA contents.

For the entire base of the experiment, the coefficient of variation ranged from 0.6% to 2.3%, which proves high homogeneity of the results. The average values of bitumen 50/70 softening point with respect to the amount of F–T synthetic wax and SAA, together with standard deviations, are shown in Figure 7.

The data in Figure 7 indicate that the softening point increases with the increase in the content of the F–T synthetic wax. This finding correlates with the decreasing penetration value due to the increased wax content. The addition of SAA produces the opposite trend; the softening point value is lower, although still higher than that obtained without the F–T synthetic wax [36]. By using both additives, it is thus possible to properly model the softening point, depending on the intended application of the mixture made with modified bitumen 50/70.

**Figure 8:**
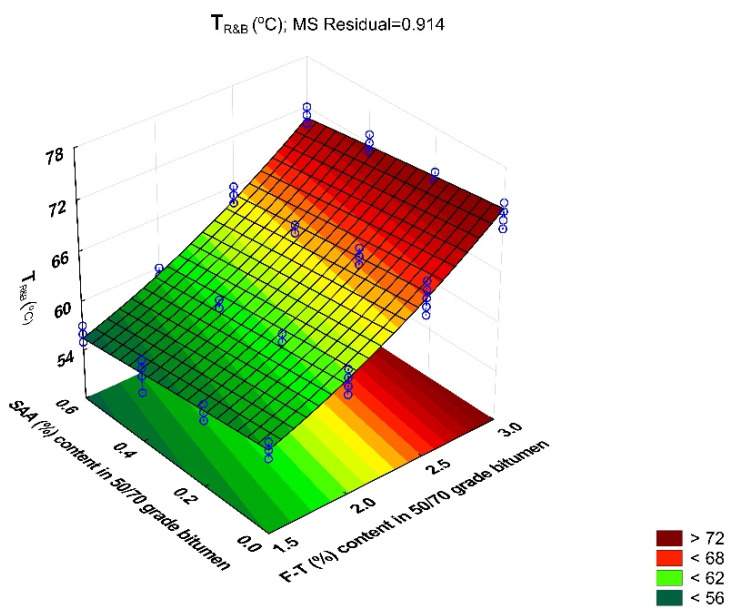
Response surface plot for T_R&B_ of bitumen 50/70 and its model.

Then, a regression model was developed to determine the strength of the relationship between the softening point of bitumen 50/70 and the contents of F–T synthetic wax and SAA. The values describing the model are summarized in Table 5. A graphical interpretation of the model is shown in Figure 8.

T_R&B_ = 54.005 − 1.063 · F–T − 3.766 · SAA − 0.066 · F–T ·SAA + 2.500 · F–T^2^ − 0.694 · SAA^2^ (7)

Analysis of the data in Table 5 allows conclusive indication that because the p_value_ is less than the assumed significance level α = 0.05 (p_value_ < 0.05), the content of F–T wax and the content of SAA have a significant impact on the bitumen 50/70 softening point. Please note that the impact of the F–T wax and SAA contents is less significant than that on penetration. Here, no synergistic effect of F–T wax and SAA was observed. The value of coefficient R^2^ = 0.974 for the T_R&B_ variable of the modified bitumen 50/70 shows a very good fit of the second-degree polynomial curve to the results obtained from the experiment.

Bitumen 50/70 has the most beneficial softening point when the content of F–T wax ranges from 2.5% to 3.0% and that of SAA is from 0% to 0.4%. A decrease in the F–T wax content reduces the softening point over the entire SAA dosing range, which affects pavement durability by making it more susceptible to permanent deformation [26,35].

**Figure 9:**
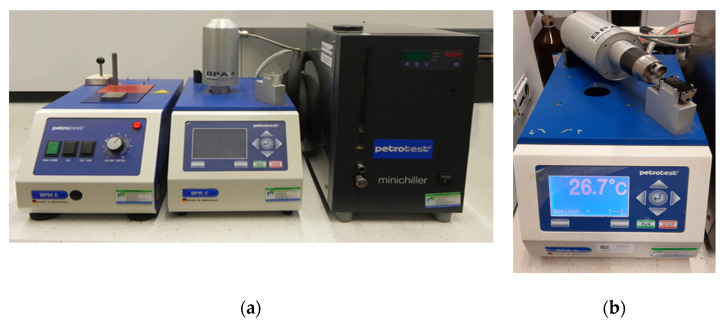
Automatic measuring device for Fraass breaking point determination (**a**), bitumen specimen (**b**) (photo by M. M. Iwański).

Another parameter analysed in this study is Fraass breaking point, determined using the automatic measuring device (Petrotest GmbH, Dahlewitz, Germany) shown in Figure 9.

**Figure 10:**
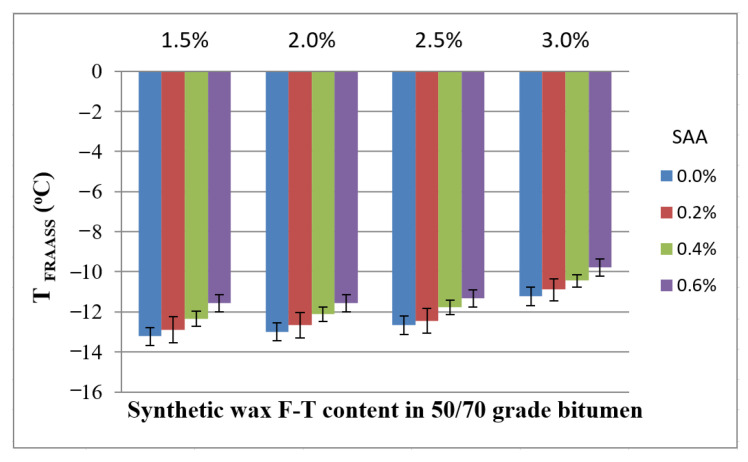
Relationship between the Fraass breaking point of bitumen 50/70 and the contents of F–T wax and SAA.

The results were characterized by high repeatability, as the coefficient of variation was between 3.7% and 7.1% throughout the entire experiment. Average values of the Fraass breaking point with respect to the F–T wax and SAA contents are given in Figure 10 together with the standard deviation.

Analysis of the data in Figure 10 allows conclusive indication that the Fraass breaking point decreases with an increasing amount of F–T wax. Bitumen 50/70 is less resistant to temperatures below zero which results from the stiffening effect from the synthetic wax at low temperature [42]. An addition of SAA has a similar, though the weaker, effect on the Fraass breaking point [36].

**Figure 11:**
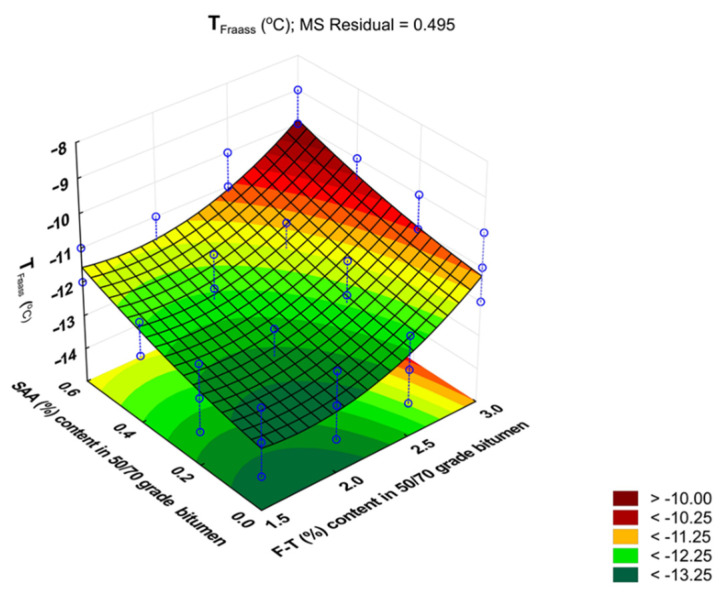
Response surface plot for Fraass breaking point of bitumen 50/70 and its model.

The results obtained for T_Fraass_ were used to develop a regression model and perform ANOVA. The values describing the model are summarized in Table 6. A graphical interpretation of the model is shown in Figure 11.

T_Fraass_ = −9.221 · F–T − 4.592 · SAA − 0.255 · F–T ·SAA + 1.305 · F–T^2^ + 1.909 · SAA^2^ (8)

Analysis of the data in Table 6 shows that it is mainly F–T wax content that has a significant impact on the Fraass point value because the p_value_ is less than the assumed significance level α = 0.05 (p_value_ < 0.05). The influence of SAA content is not significant. The value of coefficient R^2^ = 0.974 for T_Fraass_ shows a very good fit of the second-degree polynomial curve to the results obtained from the experiment.

It can be concluded that bitumen 50/70 has the most beneficial Fraass point value when the content of synthetic wax F–T is in the range 1.5% to 2.5% and the content of SAA 0.0% to 0.2%. Increasing the amount of SAA lowers the Fraass temperature of the modified binder, and consequently, the asphalt concrete made with it may have a lower resistance to low-temperature cracking than when reference bitumen 50/70 is used, as reported in [35,37].

**Figure 12:**
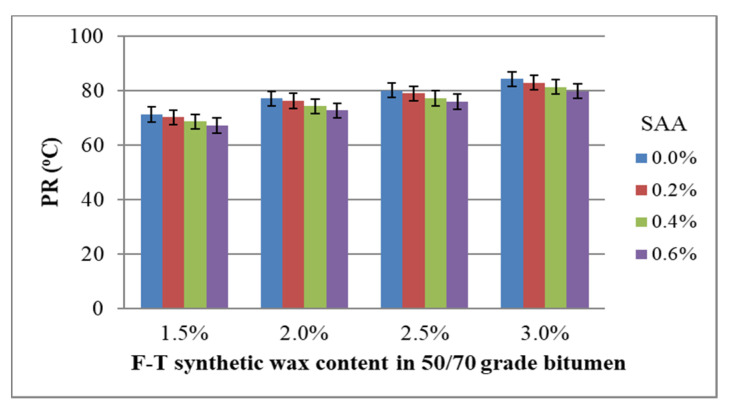
Relationship between the plasticity range of bitumen 50/70 and the contents of F–T wax and SAA.

Another parameter analysed in this study is the temperature range of plasticity. The coefficient of variation ranges from 1.3% to 2.3% for all experimental data combinations, which confirms the homogeneity of the results. Figure 12 shows the values of PR of bitumen 50/70 with respect to F–T wax and SAA contents, together with standard deviation.

Analysis of the data in Figure 12 allows for the conclusive indication that the increased content of F–T wax raises the plasticity range of bitumen 50/70. Thus, the bitumen can work properly in a wider temperature range, from low in winter too high in summer, which ensures the durability of asphalt concrete in a wide range of service temperatures. The addition of SAA produces an opposite trend; the plasticity range decreases. This results from the lowering of the bitumen’s softening point due to SAA addition. The adverse effect of SAA on the PI is reduced when F–T synthetic wax is used [36].

**Figure 13:**
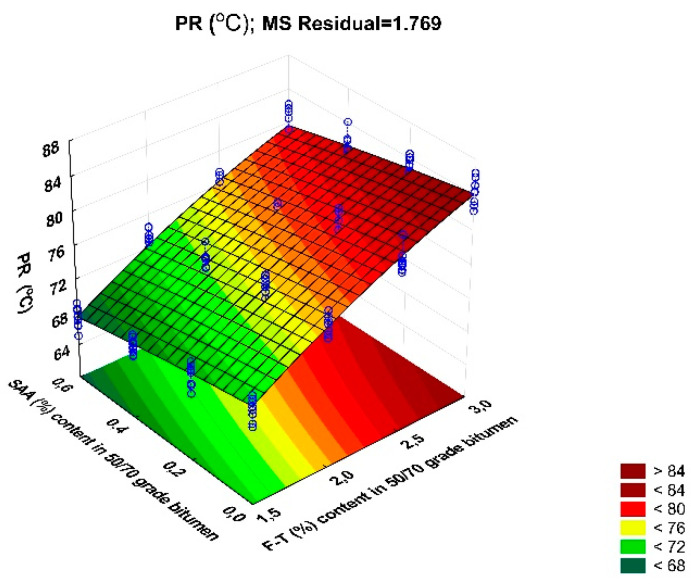
Response surface plot for plasticity range of bitumen 50/70 and its model.

The results obtained for PI were used to develop a regression model and perform ANOVA. The values describing the model are summarized in Table 7. A graphical interpretation of the model is shown in Figure 13.

PR = 76.83 + 12.373 · F–T − 4.337 · SAA − 0.129 · F–T ·SAA − 1.922 · F–T^2^ − 0.347 · SAA^2^ (9)

Analysis of the data in Table 7 allows inference of stronger impact of F–T wax on bitumen 50/70 plasticity range than that of SAA because the p_value_ for PR is less than the assumed significance level α = 0.05 (p_value_ < 0.05). Here, no synergy between F–T wax and SAA was observed. The value of coefficient R^2^ = 0.934 for the PR of the modified bitumen 50/70 shows a very good fit of the second-degree polynomial curve to the results obtained from the experiment.

Bitumen 50/70 has the most beneficial plasticity range when the content of synthetic wax F–T is in the range 2.5% to 3.0% and the content of SAA 0.0% to 0.4%. Increasing the SAA amount reduces the softening point and hence the plasticity range. As a result, concrete durability at low winter and high summer temperatures may be reduced [34,35,37].

**Figure 14:**
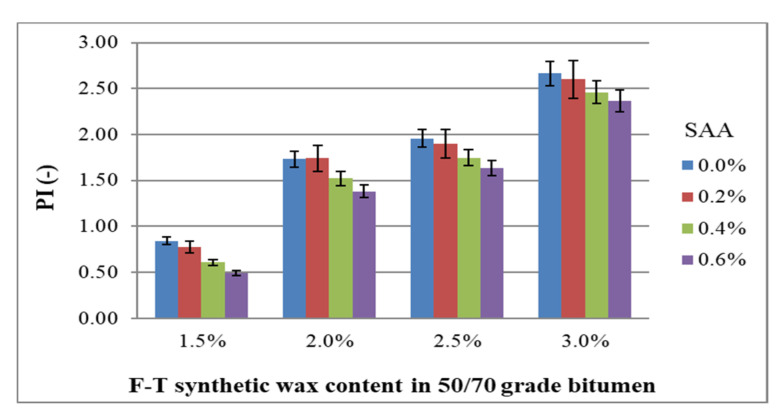
Relationship between the penetration index of bitumen 50/70 and the contents of F–T wax and SAA.

Another parameter analysed in this study is the penetration index of the modified bitumen 50/70. The coefficient of variation ranges from 2.3% to 6.3% for all experimental data combinations, which confirms the homogeneity of the results. Figure 14 shows the values of PI of bitumen 50/70 with respect to F–T wax and SAA contents, together with standard deviation.

Analysis of the data in Figure 14 indicates that the penetration index rises with the increasing content of F–T synthetic wax. Bitumen becomes stiffer. Adding SAA produces an opposite trend; the penetration index decreases. As a result, this parameter of the modified bitumen 50/70 can be modelled depending on the type and amount of chemical additive used.

**Figure 15:**
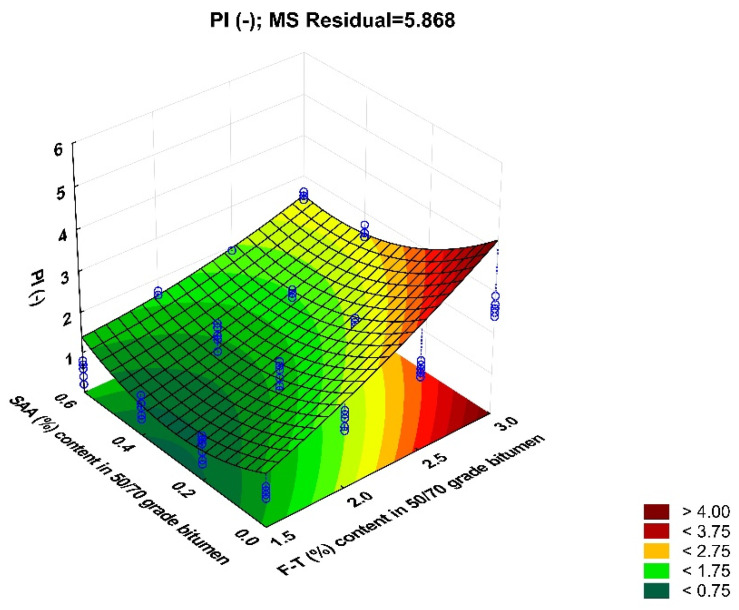
Response surface plot for and its model penetration index of bitumen 50/70 and its model.

The results obtained for PI were used to develop a regression model and perform ANOVA. The values describing the model are summarized in Table 8. A graphical interpretation of the model is shown in Figure 15.

PI = 1.487 + 2.019 · F–T − 0.817 · SAA − 0.894 · F–T ·SAA + 0.378 · F–T^2^ + 1.365 · SAA^2^ (10)

Analysis of the data in Table 8 indicates that only the F–T wax content is significant with respect to penetration index, and to a limited extent. The p_value_ is less than the assumed significance level α = 0.05 (p_value_ < 0.05).

Bitumen 50/70 has the most beneficial penetration index when the content of synthetic wax F–T is in the range 1.5% to 2.0% and the content of SAA 0.2% to 0.4%. The use of F–T synthetic wax in combination with SAA ensures that the penetration index of bitumen 50/70 is more beneficial than that obtained when only the SAA additive is used [36] or F–T synthetic wax [20].

### 3.2. Determining Dynamic Viscosity of the Binder Containing SAA and Synthetic Wax F–T

**Figure 16:**
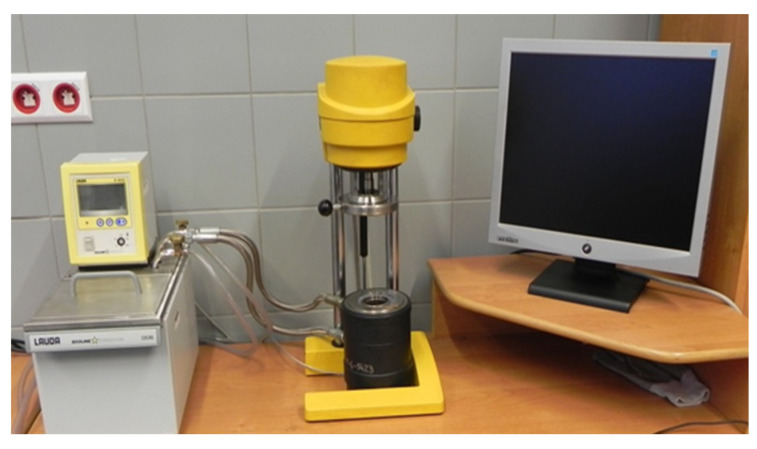
Rheotest viscometer (photo by M. M. Iwański).

Dynamic viscosity is an important rheological parameter of bitumen as it influences the physical and mechanical properties of the bitumen and the performance of bituminous layers of pavements. This parameter was determined in order to understand the influence of the SAA additive and the F–T synthetic wax on the changes occurring in the bitumen relative to the reference binder. For this purpose, samples of bitumen 50/70 were prepared, containing SAA at 0.0% to 0.6% and F–T synthetic wax at 1.5, 2.0, 2.5 and 3.0% by mass of the binder. Tests were performed as per EN 13302-2011 [49] in a Rheotest rotary viscometer (RHEOTEST Medingen GmbH, Ottendorf-Okrilla, Germany) at a shear rate of 1 s^−1^ (Figure 16)**.** Viscosity was measured at 60 °C and 90 °C using a plate–plate measurement system, while at 135 °C, the cylinder–cylinder system was used, i.e., two coaxial cylinders.

**Figure 17:**
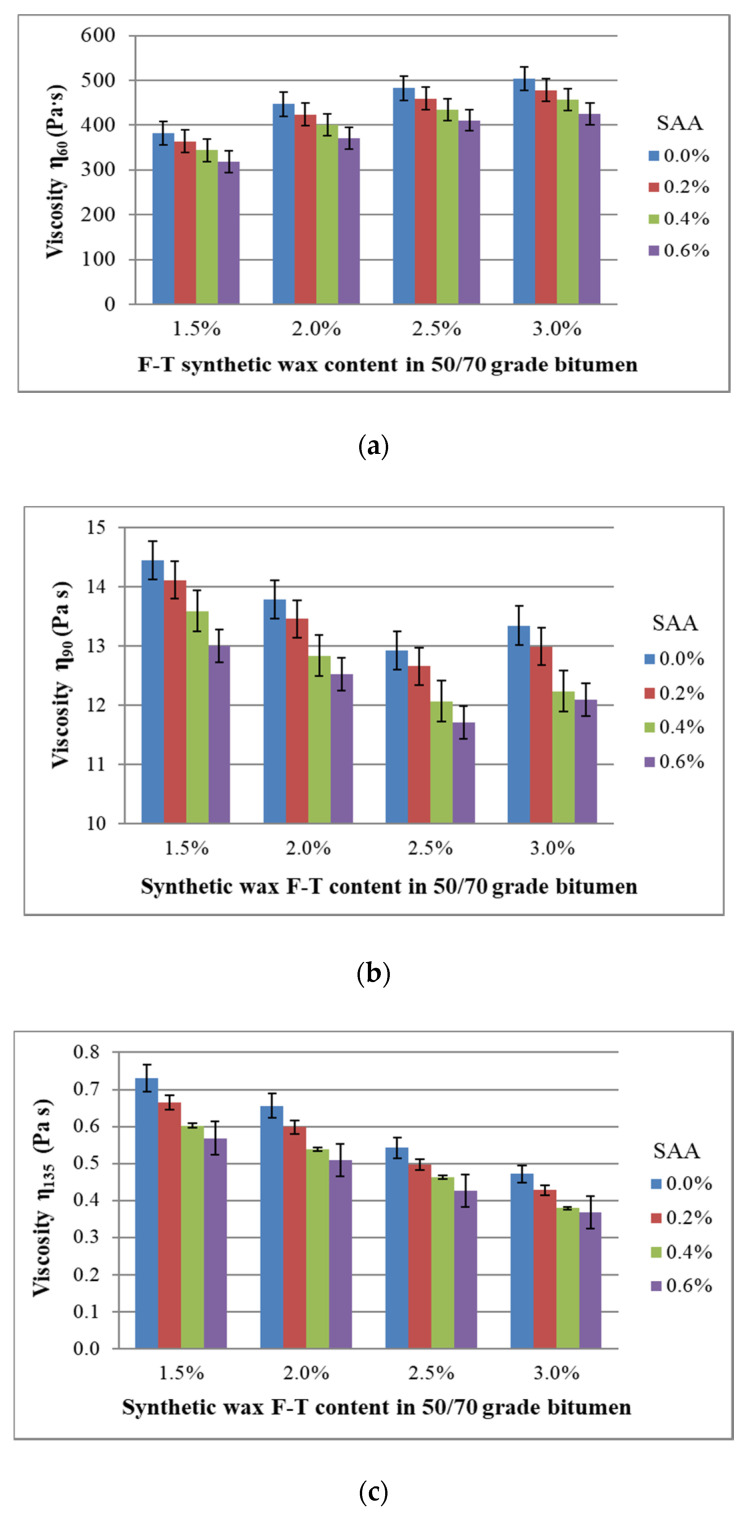
Relationship of dynamic viscosity of bitumen 50/70 containing F–T wax and SAA and temperature; 60 °C (**a**), 90 °C (**b**) and 135 °C (**c**).

The dynamic viscosity range of bitumen 50/70 with the addition of SAA and F–T synthetic wax was determined at temperatures 60 °C, 90 °C and 135 °C, corresponding to the temperatures of mixing, compaction and service of binders in pavement layers. The coefficient of variation for the viscosity with respect to the contents of F–T synthetic wax and SAA ranged from 0.5% to 2.9% for all experimental data combinations, which proves the high homogeneity of the test results. The tests were performed with the use of 9 samples for each relationship. The average values of dynamic viscosity at a given test temperature are shown in Figure 17.

The use of SAA at 60 °C increases the bitumen 50/70 viscosity, which reflects a general trend [3]. Higher viscosity binder in asphalt concrete will provide it with improved durability at high summer temperatures. The SAA impact on the viscosity at high temperatures exhibits a different trend. Although not desirable, this effect, however, confirms the possibility of modelling the binder viscosity depending on environmental conditions, e.g., when the resistance to moisture and frost of the mixture is a priority over its resistance to permanent deformation [37].

A reverse trend is observed for temperatures 90 °C and 135 °C. The viscosity of bitumen 50/70 with respect to F–T wax content decreases when the concentration of F–T wax increases. This trend is enhanced by the use of SAA addition. Here, the synergy of the F–T wax and SAA is observed, that models the viscosity of bitumen 50/70 at 90 °C and 135 °C. At high temperatures, the joint effect of both additives on bitumen viscosity is stronger than that of SAA [36] or F–T wax [43] used alone.

Please note that the quantity and test temperature of the additives used in 50/70 bitumen determine the intensity of their impact.

To check the relationship between the binder viscosity and additive contents, a viscosity model was developed and then evaluated using ANOVA and Statistica software [46]. The results obtained show the significance of F–T synthetic wax and SAA contents with respect to the dynamic viscosity of bitumen 50/70 at 60 °C, 90 °C and 135 °C because the p_value_ is less than the assumed significance level α = 0.05.

Table 9 compiles the statistical parameters describing the developed regression model of dynamic viscosity with respect to F–T wax and SAA contents.

The higher standard error (Std. Error) at SAA (%) (Q) and (L) for the viscosity relationships of the modified binder with WMA additives at 60 °C is most likely the result of a greater number of values obtained from single measurements, deviating from the average value. It should be noted that all the measurement results meet the assumptions and fall within the range of $\bar{X}$ − σ to $\bar{X}$ + σ. The coefficient of variation of the viscosity measurements at 60 °C ranges from 0.5% to 1.9%, with standard deviation values from 2.38 MPa to 7.34 MPa. This proves the high homogeneity of the test results. Obtaining a significant number of measurements at the limits of acceptable result interval may result from the phase change of WMA components in the bitumen at this temperature. At 60 °C, F–T wax is already in the solid phase—it crystallizes and the SAA still remains in the liquid phase, causing the bitumen to plasticize. Large phase differentiation of additives has an impact on the results of single viscosity measurements. At 90 °C and 135 °C, both the F–T synthetic wax and the SAA are in the liquid phase, ensuring the high homogeneity of the binder. Consequently, the results of single measurements are very close to the average value and SD has very low values.

Statistical analysis results indicate a very good fit of the model because p_value_ < 0.05 in all cases analysed. The values of coefficient R^2^ for dynamic viscosity of bitumen 50/70 with respect to the additive type and test temperature (60 °C, 90 °C and 135 °C) are 0.991, 0.809 and 0.985, respectively, which shows a very good fit of the model to the results obtained from the experiment.

The data obtained from statistical analysis were used to develop the models expressing the relationship between the dynamic viscosity of the binder at 60 °C, 90 °C, and 135 °C with respect to the F–T wax and SAA contents:

η_60_ = 84.557 + 259.376 · F–T − 31.944 · SAA − 12.975 · F–T ·SAA − 39.916 · F–T^2^ -31.944 · SAA^2^ (11)

η_90_ = 20.186 − 5.207 · F–T − 2.396 · SAA + 0.117 · F–T ·SAA + 0.964 · F–T^2^ − 0.191 · SAA^2^ (12)

η_135_ = 0.521 − 0.235· F–T − 0.134 · SAA + 0.031 · F–T ·SAA − 0.004 · F–T^2^ + 0.029 · SAA^2^ (13)

**Figure 18:**
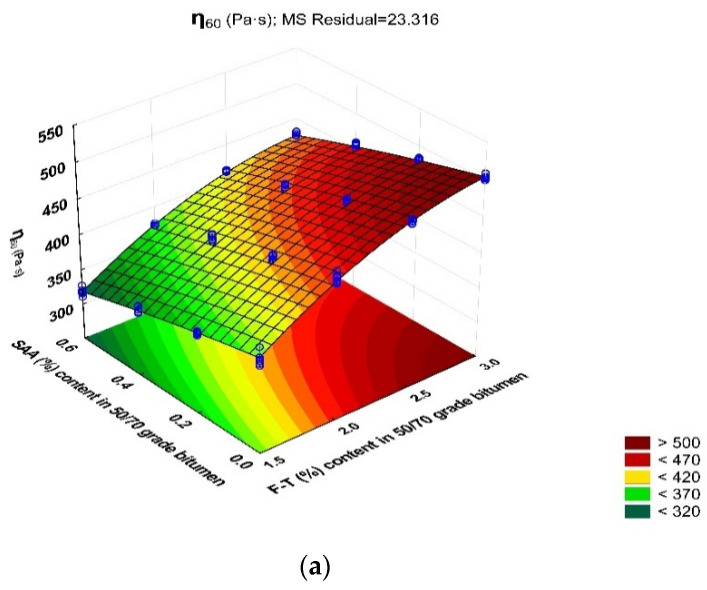

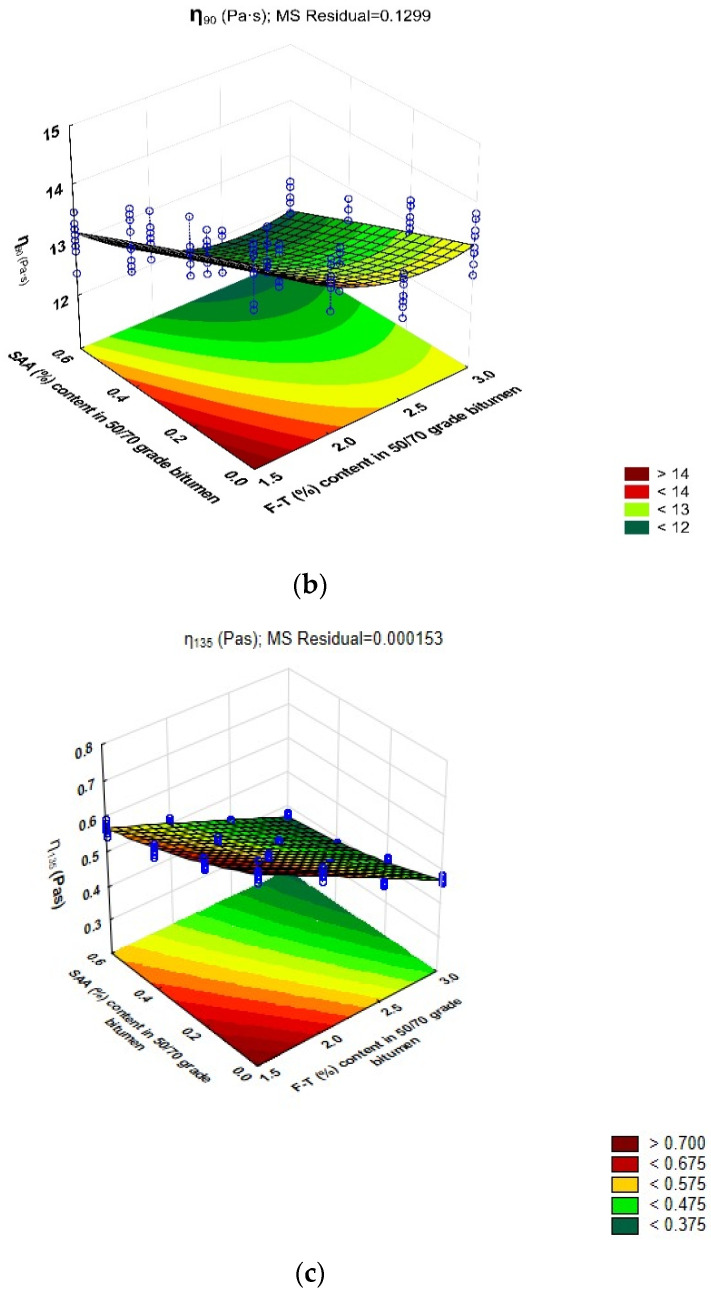
Response surface plots for dynamic viscosity of bitumen 50/70 with respect to F–T synthetic wax content and SAA content at 60 °C (**a**), 90 °C (**b**) and 135 °C (**c**).

A graphical interpretation of the dynamic viscosity change with respect to F–T wax and SAA contents is represented as response surface plots in Figure 18.

The intensity of the F–T wax and SAA effect on dynamic viscosity depends on test temperature. At 60 °C, the F–T wax content in the range of 0.2% to 0.6% and the SAA content of 0.0% to 0.4% are most beneficial. Increasing the concentration of SAA leads to a decrease in dynamic viscosity, which is not advisable for the proper in-service performance of bituminous mixtures.

This trend changes at test temperature increased to 90 °C. When 2.0% to 3.0% of F–T synthetic wax and 0.2% to 0.6% of SAA is used, the dynamic viscosity of bitumen 50/70 is low. The trend remains the same at 135 °C, where most beneficial test results are obtained with 2.5% to 3.0% F–T synthetic wax and 0.2% to 0.6% SAA. As the result of synergy, the use of both additives has a more favourable effect on bitumen 50/70 viscosity in the temperature range under analysis than that obtained from either of them alone [3,32,36]. Binders with these contents of the additives will provide mixtures that can be prepared at the lower temperature, but the required durability parameters will still be maintained.

### 3.3. Foamed Bitumen Properties

**Figure 19:**
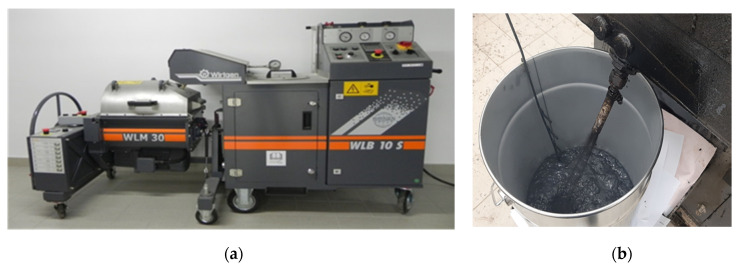
WLB-10 foamed bitumen plant (**a**) and a adapter for determining foamed bitumen characteristics (**b**).

The bitumen was foamed using the WLB-10S (Wirtgen GmbH, Windhagen, Germany) foamed additionally equipped with a mixing unit for the production of bituminous mixtures (WLM-30) (Figure 19).

The F–T synthetic wax was used at 1.5%, 2.0%, 2.5% and 3.0% as recommended in [42,43] The surface-active agent SAA was added at 0.2%, 0.4% and 0.6% relative to the mass of the binder. The SAA amount of 0.6% relative to the bitumen mass was adopted as the maximum content because this value is recommended by the manufacturer as the boundary value between the hot and cold mixtures [36].

**Figure 20:**
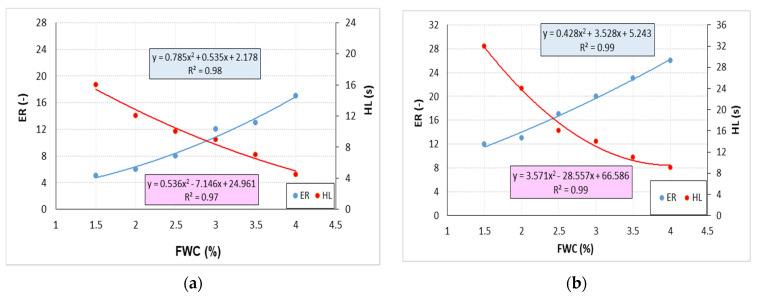

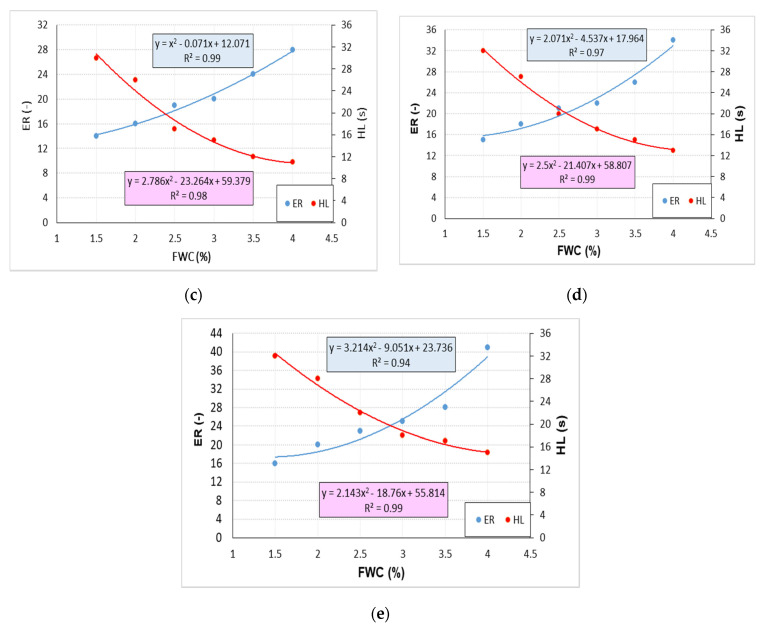
Bitumen 50/70 foam characteristics (**a**), bitumen 50/70 + 0.6% SAA + 1.5% F–T (**b**), bitumen 50/70 + 0.6% SAA+ 2.0% F–T (**c**), bitumen 50/70 + 0.6% SAA + 2.5% F–T (**d**), bitumen 50/70 + 0.6% SAA + 3.0% F–T (**e**).

First, the classic foam parameters (ER, HL) were characterized for the reference bitumen 50/70 and the bitumen modified with 0.6% SAA and 1.5%, 2.0%, 2.5% and 3.0% of F–T wax with the average value from 9 determinations (Figure 20). Foaming water content ranged from 1.5% to 4.0% used at 0.5% steps, which was in line with the general practice [16,18]. The foam parameters for each of the considered combinations of bitumen 50/70 with chemical additives were determined in a similar way and then analysed.

**Figure 21:**
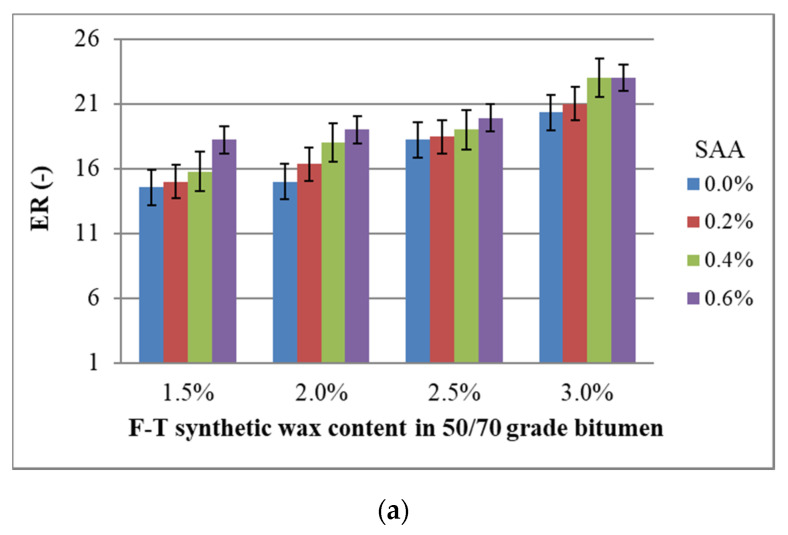

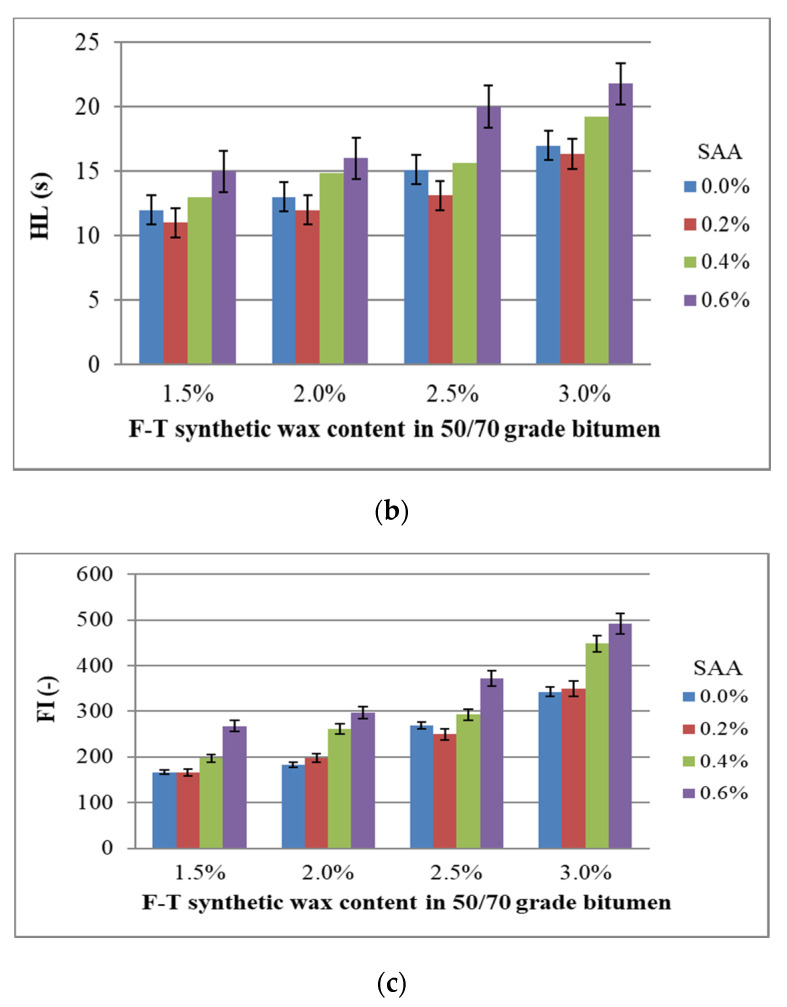
Bitumen 50/70 foam parameters for, ER (**a**), HL (**b**), FI (**c**).

The results of testing the foam characteristics of bitumen 50/70 while ensuring the optimum amount of foaming water with respect to the addition of chemical additives used are shown in Figure 21.

The use of F–T synthetic wax and SAA increases the bitumen 50/70 foam characteristics. An increase of nearly 80% is observed with the F–T wax content of 3.0%. The addition of SAA enhances this trend. As a result of using both additives, bitumen 50/70 has much higher foaming parameters than when either of the additives is used alone [18,20,36]. At 0.2% SAA, a decrease in HL of the bitumen foam is noted. This phenomenon may be caused by an adverse interaction of both additives and will be subject to detailed chemical analysis in the next stage of the research.

In order to determine the dependence of the bitumen 50/70 foam parameters in terms of the additives used, a significance test was performed using the analysis of variance (ANOVA) and Statistica software [46]. The results show clearly that SAA and F–T wax content is a significant factor because the p_value_ is less than the assumed significance level α = 0.05.

The results obtained for dynamic viscosity were used to develop a regression model. The values describing the model are summarized in Table 10.

The models developed to evaluate the relationships between the foam characteristics and F–T wax and SAA contents have the following form:

ER = 17.746 + 5.891 · F–T + 3.075 · SAA − 0.605 · F–T ·SAA + 1.968 · F–T^2^ + 0.468 · SAA^2^ (14)

HL = 13.739 + 5.850 · F–T + 4.300 · SAA + 1.080 · F–T ·SAA + 1.562 · F–T^2^ + 4.125 · SAA^2^ (15)

FI = 242.699· + 206.231 · F–T + 123.335 ·SAA + 25.998 · F–T ·SAA + 86.097 · F–T^2^ + 64.360 · SAA^2^ (16)

**Figure 22:**
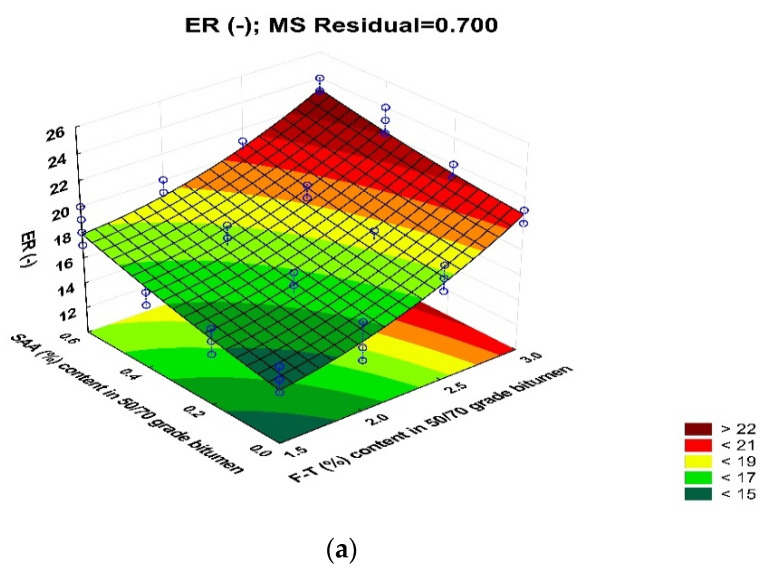

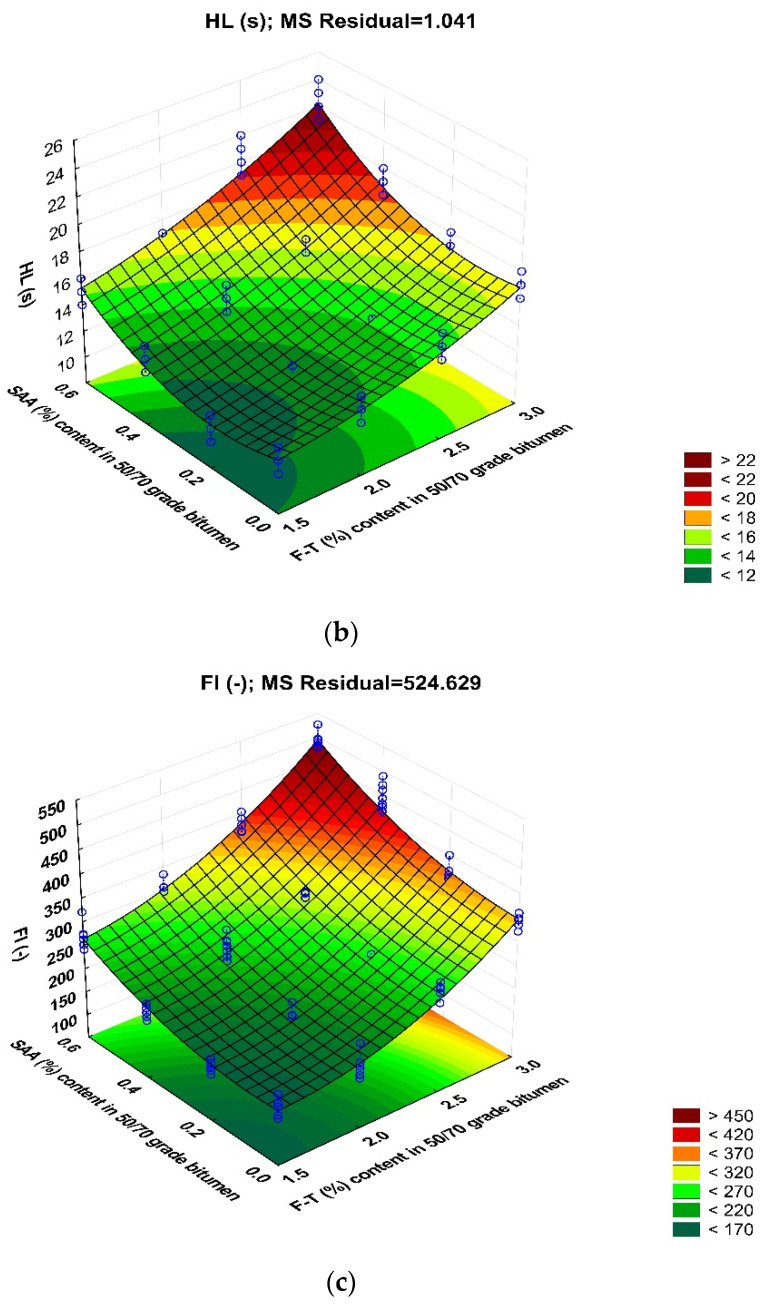
Relationships between bitumen 50/70 foam parameters and F–T wax and SAA contents; maximum expansion—ER (**a**), half-life—HL (**b**) and foam index—FI **(c).**

A graphical interpretation of the change in bitumen 50/70 foam characteristics with respect to the F–T wax and SAA content is shown as response surface plots in Figure 22.

Analysis of the bitumen 50/70 foam characteristics with respect to F–T wax and SAA contents used shows an evident positive effect and a significant impact because the p_value_ is less than the assumed significance level α = 0.05. Due to the synergy effect in terms of lowering the bitumen viscosity, the use of both additives provides much better foaming parameters for bitumen 50/70 than those obtained when either of the additives is used alone [18,20,36]. This binder type will thus have a positive effect on ensuring the required resistance to water, frost, and permanent deformation of asphalt concrete [26,30,34].

Reliability of the test results is confirmed by high values of R^2^, which is 0.905 for ER, 0.856 for HL, and 0.945 for FI. It is important to emphasize the synergy between both additives with respect to ER, HL, and FI. As a result, the binder will more efficiently coat the aggregate particles during the production of the bituminous mix, ensuring its durability and proper performance in the pavement.

The developed models of bitumen 50/70 foam characteristics helped determine the required amount of F–T synthetic wax and SAA, depending on its intended use. Depending on the needs, the composition of the modified binder (foamed bitumen) can be established with the respect of mineral material temperature for the purpose of making a cold mixture (CMA—cold mix asphalt) in terms of the temperature of the mineral material (Table 11) or the technology in which it is to be used—Table 12.

Based on the developed dependency models (6, 7, 8, 9, 10, 11, 12, 13, 14, 15, 16) of the tested parameters of bitumen 50/70, it is possible to simultaneously determine the significant properties of the modified binder and analyse their effect on the properties of the bituminous mixture.

### 3.4. SAA and F–T Synthetic Wax Contents for Optimized Bitumen Properties

Statistica program [46] was used to determine the recommended contents of SAA and F–T synthetic wax for the optimized properties of bitumen 50/70 intended for HWMA mixture.

The following significant parameters of bitumen 50/70 containing 0%, 0.2%, 0.4% and 0.6% SAA and 1.5%, 2.0%, 2.5% and 3.0% F–T synthetic wax were analysed:

- Penetration at 25 °C (Pen),
- Softening point (T_PiK_);
- Fraass braking point (T_Fraass_);
- Penetration index (PI);
- Dynamic viscosity at 60 °C (η_60_), 90 °C (η_90_) and 135 °C (η_135_);
- Maximum expansion ratio of bitumen foam (ER);
- Half-life of bitumen foam (HL);
- Foam index (FI).

Table 13 compiles the characteristics of the models describing the relationships between the binder parameters and the contents of F–T wax and SAA.

Statistical parameters in Table 13 clearly indicate a very significant impact on the process of optimizing the properties of bitumen 50/70 in terms of the additives used in the tests (p_value_ < p = 0.05). The high values of the coefficient of R^2^ from 0.809 to 0.985, confirm the reliability of the optimization results. The R^2^ coefficient was low only in the case of the PI parameter, which can be attributed to the fact that it is a calculation parameter dependent on two test parameters of the binder. As shown in Table 13, this parameter not significantly influences the binder optimization process.

In order to evaluate the suitability of the binder, criteria were adopted for the analysed parameters, according to which their most desirable values were given a desirability index equal to 1, and the least desirable values were given the value of 0. Intermediate values had desirability indices ranging from 0 to 1 in a linear relationship. The optimization procedure used was described in detail in [60]. The following criteria were used for individual binder parameters:

- Penetration at 25 °C (max—0, min—1);
- Softening point T_R&B_ (max—1, min—0);
- Fraass breaking point T_Fraass_ (max—0, min—1);
- Penetration index PI (max—1, min—0);
- Dynamic viscosity at a temperature of η_60_ (max—1, min—0);
- Dynamic viscosity at temperatures of η_90_ and η_135_ (max—0, min—1);
- Maximum expansion ratio of the foam ER (max—1, min—0);
- Half-life of the foam HL (max—1, min—0);
- Foam index FI (max—1, min—0);
- Plasticity range PR (max—1, min—0).

It should be noted that, depending on the intended use of foamed bitumen, dedicated criteria can be used to indicate the quantity of chemical additive ensuring appropriate binder properties for a specific type of bituminous mixture (e.g., deep cold recycling, WMA mixture, etc.).

**Figure 23:**
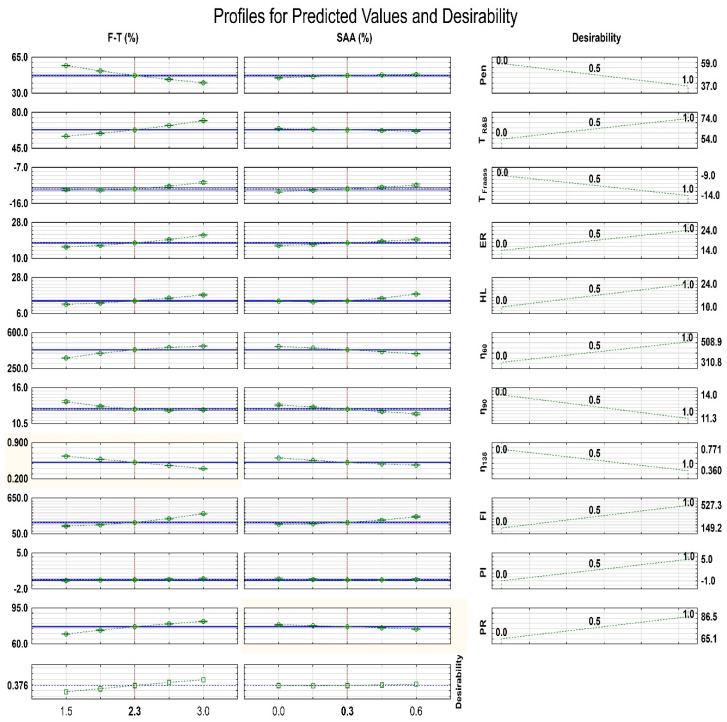
Determining the optimum contents of F–T wax and SAA for optimized properties of bitumen 50/70.

Then desirability function values were calculated using Statistica [56]. The results are summarized in Figure 23 as profiles for the predicated and desirability values.

The optimization results show that the addition of F–T synthetic wax and SAA had different effects on the assessed properties of the binder in terms of its utility. F–T synthetic wax had a positive effect on the softening point and penetration. A different effect on these parameters was observed when SAA was added. Both chemical additives had a negative effect on the Fraass breaking point, but this effect, as found in detailed analyses, should not be considered very important. The dynamic viscosities measured at 90 °C and 135 °C decreased after the incorporation of both additives in greater amounts, which in turn contributes to the improved coating of aggregate by the binder and more efficient mixture production process. At 60 °C, the addition of F–T synthetic wax increased viscosity, and the effect of SAA was less significant as a decrease was observed. The penetration index after the incorporation of both additives remained constant. The range of plasticity changed after adding the F–T synthetic wax to the binder in the range 1.5% to 2.0%, and a further increase in its concentration did not change it significantly. No significant influence of SAA on plasticity range was observed. The analysis of the binder foam parameters (ER, HL, FI) showed a significant influence of both the F–T wax and SAA leading to improved workability and homogeneity of the mixture.

The overall evaluation of the effects of F–T synthetic wax and SAA on the properties of bitumen 50/70 intended for use in bituminous mixtures with foamed bitumen results from the calculated desirability profile.

The desirability analysis of the approximated values shows that the recommended SAA content used as an additive to 50/70 bitumen is 0.3% and the synthetic wax F–T content is 2.3% relative to bitumen mass. In this case, the binder consisting of bitumen 50/70 and the analysed chemical additives will obtain optimum parameters, which should significantly ensure the proper mixture production and pavement structure, and guarantee pavement durability. From the approximated values it was found that the recommended SAA content used as an additive to bitumen 50/70 is 0.3% and that of the F–T synthetic wax is 2.3% relative to the mass of the bitumen. In this case, the binder consisting of bitumen 50/70 and the analysed chemical additives will obtain optimum parameters, which should guarantee proper mixture manufacture, pavement structure, and pavement durability.

## 4. Conclusions

The use of F–T synthetic wax and surface active agent SAA has a significant effect on the general properties of bitumen 50/70 and its foam characteristics. The following conclusions were reached based on the data presented in this article:

- The use of F–T synthetic wax increases the value of basic characteristics of 50/70 bitumen such as penetration at 25 °C and softening point. However, the SAA addition shows the opposite trend.
- F–T synthetic wax and SAA at the test temperature of 135 °C and 90 °C has a beneficial effect on dynamic viscosity; the binder with the lowered value of dynamic viscosity can be applied to mixtures produced at lower temperatures than the conventional temperature. At 60 °C, the synthetic wax increases the viscosity of bitumen 50/70, whereas the use of SAA decreases the value of this parameter.
- Increasing the content of F–T synthetic wax and SAA leads to higher values of bitumen 50/70 foam characteristics owing to the synergy that occurs between these two additives.
- The models of bitumen 50/70 foam characteristics as a function of F–T wax and SAA contents allow the selection of adequate binder composition for dedicated applications or technologies (the type of works, temperature range).
- The optimum content of the additives was found to be 2.3% for F–T synthetic wax and 0.3% for SAA as determined through the bitumen 50/70 optimization process. The binder containing these quantities of the additives ensures the most beneficial foam characteristics and the required level of standard parameters.

In summary, the use of the F–T synthetic wax additive and the SAA additive will have a positive effect on bitumen 50/70 foaming and thus on its application with various technologies, including HWMA. At the same time, the existing mechanical characteristics of the bituminous mix will be maintained at a high level. Modification of bitumen 50/70 with both additives should ensure proper mixture production and incorporation processes at reduced temperatures.

## Figures and Tables

**Table 1:** Basic properties of 50/70 bitumen [36].

Property	Test Method	Unit of Measure	Result
Penetration at 25 °C	EN 1426 [46]	0.1 mm	65.9
Softening point T_R&B_	EN 1427 [47]	°C	50.4
Fraass breaking point	EN 12593 [48]	°C	−15.1
Dynamic viscosity at: 60 °C 90 °C 135 °C	EN 13302 [49]	Pa∙s	372.9 14.0 0.649

**Table 2:** Characteristics of the synthetic wax F–T [50,51].

Property	Unit of Measure	Value
Colour	-	white, yellowish
Flash point	°C	285
Congening point	°C	95
Density at 25 °C	Mg/m^3^	0.9
Viscosity at 135 °C	cSt	12
Molecular mass	g/mol	about 1000

**Table 3:** Characteristics of the surface-active agent (SAA) [36,52,53].

Property	Unit of Measure	Value
Appearance	-	Brown viscous liquid
Density at 20 °C	Mg/m^3^	0.98
Pour point	°C	<0
Viscosity at 20 °C	mP	3000
Viscosity at 50 °C	mP	400
Amine index	mg HCl/g	159–185
Acid index	mg KOH/g	<10
Freezing point	°C	<0
Flash point (open flame)	°C	>218

**Table 4:** Evaluation of the significance (ANOVA) of the factorial impact (F–T wax and SAA) on Pen.

Effect	Regresion Coefficients	Std. Error	p_value_
Variable: Pen (0,1 mm); R^2^ = 0.974; R^2^ adj:0.972; MS Residual = 1.136			
Intercept	80.873	1.799	<0.001
(1) F–T (%) (L)	−21.112	1.621	<0.001
F–T (%) (Q)	2.305	0.355	<0.001
(2) SAA (%) (L)	12.529	2.119	<0.001
SAA (%) (Q)	−7.465	2.21	<0.001
1L × 2L	−1.188	0.710	0.097

Where: Q—quadratic; L—linear.

**Table 5:** Evaluation of the significance (ANOVA) of the factorial impact (F–T wax and SAA) on T_R&B._

Effect	Regresion Coefficients	Std. Error	p_value_
Variable: T_R&B_ (°C); R^2^ = 0.9746; R^2^ adj:0.973; MS = 0.913			
Intercept	54,005	1.613	<0.001
(1) F–T (%)(L)	−1.063	1.453	0.466
F–T (%)(Q)	2.500	0.318	<0.001
(2) SAA (%)(L)	−3.766	1.899	<0.001
SAA (%)(Q)	−0.694	1.991	0.728
1L × 2L	−0.066	0.637	0.917

Where: Q—quadratic; L—linear.

**Table 6:** Evaluation of the significance (ANOVA) of the factorial impact (F–T wax and SAA) on T_Fraass._

Effect	Regresion Coefficients	Std. Error	p_v__alue_
Variable: TFraass (°C); R^2^ = 0.64902; R^2^Adj: 0.636; MS Residual = 0.494			
Intercept	−9.221	1.186	<0.001
(1) F–T (%) (L)	−4.592	1.069	<0.001
F–T (%) (Q)	1.305	0.234	<0.001
(2) SAA (%) (L)	1.915	1.398	0.173
SAA (%) (Q)	1.909	1.465	0.195
1L × 2L	−0.255	0.468	0.587

Where: Q—quadratic; L—linear.

**Table 7:** Evaluation of the significance (ANOVA) of the factorial impact (F–T wax and SAA) on PR.

Effect	Regresion Coefficients	Std. Error	p_v__alue_
Variable: PR; R^2^ = 0.934; R^2^Adj: = 0.931 MS Residual = 1.769			
Intercept	76.833	0.225	<0.001
(1) F–T (%) (L)	12.373	0.297	<0.001
F–T (%) (Q)	−1.922	0.498	<0.001
(2) SAA (%) (L)	−4.337	0.297	<0.001
SAA (%) (Q)	−0.347	0.498	0.486
1L × 2L	−0.129	0.399	0.745

Where: Q—quadratic; L—linear.

**Table 8:** Evaluation of the significance (ANOVA) of the factorial impact (F–T wax and SAA) on PI.

Effect	Regresion Coefficients	Std. Error	p_v__alue_
Variable: PI; R^2^ = 0.127; R^2^Adj: = 0.095; MS Residual = 5.868			
Intercept	1.487	0.410	<0.001
(1) F–T (%) (L)	2.019	0.541	<0.001
F–T (%) (Q)	0.378	0.908	0.677
(2) SAA (%) (L)	−0.817	0.541	0.133
SAA (%) (Q)	1.365	0.908	0.135
1L × 2L	−0.894	0.726	0.220

Where: Q—quadratic; L—linear.

**Table 9:** Parameters of the regression model for dynamic viscosity variables η_60_, η_90_, η_135_ of bitumen 50/70 with respect of F–T wax and SAA contents.

Effect	Regresion Coefficient	Std. Error	p_value_
**Variable: η_60_ (Pas); R^2^ = 0.991; R^2^Adj: 0.991; MS Residual = 23.316**			
Intercept	84.557	8.149	<0.001
(1) F–T (%) (L)	259.376	7.342	<0.001
F–T (%) (Q)	−39.916	1.609	<0.001
(2) SAA (%) (L)	−71.099	9.599	<0.001
SAA (%)(Q)	−31.944	10.059	<0.001
1L × 2L	−12.975	3.219	<0.001
**Variable: η_90_ (Pas); R^2^ = 0.809; R^2^Adj: 0.802; MS Residual = 0.129**			
Intercept	20.186	0.608	<0.001
(1) F–T (%) (L)	−5.207	0.548	<0.001
F–T (%) (Q)	0.963	0.120	<0.001
(2) SAA (%) (L)	−2.396	0.716	<0.001
SAA (%) (Q)	−0.190	0.750	0.799
1L × 2L	0.116	0.240	0.628
**Variable: η_135_ (Pas); R^2^ = 0.985; R^2^Adj: 0.985; MS Residual=0.001**			
Intercept	0.520	0.002	<0.001
(1) F–T (%) (L)	−0.235	0.003	<0.001
F–T (%) (Q)	−0.003	0.004	0.405
(2) SAA (%) (L)	−0.134	0.003	<0.001
SAA (%) (Q)	0.029	0.005	<0.001
1L × 2L	0.031	0.004	<0.001

Where: Q—quadratic L—linear.

**Table 10:** Parameters of the model expressing the relationships between ER, HL, FI and the F–T wax and SAA content in bitumen 50/70.

Response	Effect	Regression Coefficient	Std. Error	p_value_
ER (-) R^2^ = 0.905 R^2^Adj: 0.901 MS Res. = 0.700	Intercept	17.746	0.141	<0.001
	(1) F–T (%) (L)	5.891	0.187	<0.001
	F–T (%) (Q)	1.968	0.313	<0.001
	(2) SAA (%) (L)	3.075	0.187	<0.001
	SAA (%) (Q)	0.468	0.313	0.137
	L × 2L	−0.605	0.251	0.017
HL (s) R^2^ = 0.893 R^2^Adj: 0.889; MS Res. = 1.041	Intercept	13.739	0.172	<0.001
	(1) F–T (%) (L)	5.850	0.228	<0.001
	F–T (%) (Q)	1.562	0.382	<0.001
	(2) SAA (%) (L)	4.300	0.228	<0.001
	SAA (%) (Q)	4.125	0.382	<0.001
	1L × 2L	1.080	0.306	<0.001
FI (%) R^2^ = 0.945; R^2^Adj: 943; MS Res. = 524.628	Intercept	242.699	3.876	<0.001
	(1) F–T (%) (L)	206.231	5.121	<0.001
	F–T (%) (Q)	86.097	8.589	<0.001
	(2) SAA (%) (L)	123.335	5.121	<0.001
	SAA (%) (Q)	64.360	8.589	<0.001
	1L × 2L	25.998	6.871	<0.001

Where: Q—quadratic; L—linear.

**Table 11:** Minimum values of foamed bitumen parameters for CMA [59].

Parameter	Temperature of Mineral Material	
	10 °C to 15 °C	Above 15 °C
Expansion ratio ER (-)	10	8
Half-life HL (s)	6	6

**Table 12:** Required values of bitumen foam parameters by use of the binder [16].

Type of Mixture with Foamed Bitumen	Required Minimum Values		
	ER	HL (s)	FI (s)
Surface dressing	10	30	131
CMA	15	15	164
Surface recycling	17	13	180
WMA			

**Table 13:** Parameters of the models characterizing the variables subjected to optimization.

Dependent Variable	SS Test for the Full Fodel with Respect to SS for Residual					
	Multicrit. R	Multicrit. R^2^	Adjusted R^2^	SS Model	MS Model	p_value_
Pen (0.1 mm)	0.986	0.973	0.972	5810.8	1162.15	<0.001
T_R&B_ (°C)	0.987	0.974	0.973	4842.8	968.56	<0.001
T_Fraass_ (°C)	0.805	0.649	0.636	126.2	25.25	<0.001
ER (-)	0.951	0.904	0.901	916.5	183.31	<0.001
HL (s)	0.945	0.893	0.889	1205.6	241.11	<0.001
η_60_ (Pas)	0.995	0.991	0.991	376,660	75,332.12	<0.001
η_90_ (Pas)	0.899	0.809	0.802	76.2	15.24	<0.001
η_135_ (Pas)	0.992	0.985	0.985	1.5	0.30	<0.001
FI (-)	0.972	0.945	0.943	1,244,535	248,907	<0.001
PI (-)	0.356	0.127	0.095	118.0	23.6	<0.001
PR (°C)	0.966	0.934	0.931	3466.0	693.2	<0.001

## Data Availability

Data sharing is not applicable to this article.

## References

[1] Leng Z., Gamez A., Al-Qadi I.L. (2014). Mechanical property characterization of warm-mix asphalt prepared with chemical additives. J. Mater. Civ. Eng..

[2] Wing-gun W., Gang L. (2009). Analysis of the effect of wax content on bitumen under performance grade classification. Constr. Build. Mater..

[3] Iwański M., Mazurek G. The influence of the low-viscosity modifier on viscoelasticity behavior of the bitumen at high operational temperature. Proceedings of the 8th International Conference Environmental Engineering.

[4] Król J., Kowalski K., Radziszewski P. (2015). Rheological behavior of n-alkane modified bitumen in aspect of Warm Mix Asphalt technology. Constr. Build. Mater..

[5] Ai C., Li O.J., Qiu Y. (2015). Testing and assessing the performance of a new warm mix asphalt with SMC. J. Traffic Transp. Eng..

[6] Hurley G., Prowell B. (2005). Evaluation of Sasobit for Use in Warm Mix Asphalt.

[7] Sengoz B., Topa A., Gorkem C. (2013). Evaluation of natural zeolite as warm mix asphalt additive and its comparison with other warm mix additives. Constr. Build. Mater..

[8] Woszuk A., Franus W. (2016). Properties of the Warm Mix Asphalt involving clinoptilolite and Na-P1 zeolite additives. Constr. Build. Mater..

[9] Chomicz-Kowalska A., Maciejewski K., Iwański M.M. (2020). Study of the Simultaneous Utilization of Mechanical Water Foaming and Zeolites and Their Effects on the Properties of Warm Mix Asphalt Concrete. Materials.

[10] Jenkins K.J., de Groot J.L.A., Van de Ven M.F.C., Molenaar A. Half-warm foamed bitumen treatment, a new process. Proceedings of the 7th Conference on Asphalt Pavements for Southern Africa.

[11] Yu X., Wang Y., Luo T. (2013). Impacts of water content on rheological properties and performance-related behaviours of foamed war-mix asphalt. Constr. Build. Mater..

[12] Woszuk A., Zofka A., Bandura L., Franus W. (2017). Effect of zeolite properties on asphalt foaming. Constr. Build. Mater..

[13] Barthel W., Marchand J., Von Devivere M. (2004). Warm Mix Asphalt by adding a synthetic zeolite. Proceedings of the Third Eurasphalt and Eurobitume Conference.

[14] Van De Ven M.F.C., Jenkins K.J., Voskuilen J.L.M., Van De Beemt R. (2007). Development of (half-) warm foamed bitumen mixes: State of the art. Int. J. Pavement Eng..

[15] Muthen K.M. (2009). Foamed Asphalt Mixes. Mix Design Procedure.

[16] Jenkins K.J. (2000). Mix Design Considerations for Cold and Half-Warm Bituminous Mixes with Emphasis on Foamed Bitumen. Ph.D. Thesis.

[17] Wirtgen (2012). Wirtgen Cold Recycling Technology.

[18] Iwański M., Chomicz-Kowalska A. (2014). Evaluation of the effect of using foamed bitumen and bitumen emulsion in cold recycling technology. Proceedings of the 3rd International Conference on Transportation Infrastructure (ICTI), Sustainability, Eco-Efficiency and Conservation in Transportation Infrastructure Asset Management.

[19] Buczyński P., Iwański M. (2017). Inactive Mineral Filler as a Stiffness Modulus Regulator in Foamed Bitumen-Modified Recycled Base Layers. IOP Conf. Ser. Mater. Sci. Eng..

[20] Iwański M., Chomicz-Kowalska A., Maciejewski K. (2015). Application of synthetic wax for improvement of foamed bitumen parameters. Constr. Build. Mater..

[21] Saleh M. (2006). Characterization of Foam Bitumen Quality and the Mechanical Properties of Foam Stabilized Mixes.

[22] Mallick R.B., Hendrix G. (2004). Use of foamed asphalt in recycling incinerator ash for construction of stabilized base course. Resour. Conserv. Recycl..

[23] Iwański M., Cholewińska M. (2014). Application of the foamed bitumen and bitumen emulsion to the road base mixes in the deep cold recycling technology. Balt. J. Road Bridge Eng..

[24] Iwański M., Buczyński P., Mazurek G. (2016). The use of gabbro dust in the cold recycling of asphalt paving mixes with foamed bitumen. Bull. Pol. Acad. Sci. Tech. Sci..

[25] Yan J., Ni F., Yang M., Li J. (2010). An experimental study on fatigue properties of emulsion and foam cold recycled mixes. Constr. Build. Mater..

[26] Mrugała J., Iwański M. (2015). Resistance to permanent deformation of asphalt concrete with F-T wax modified foamed bitumen. 7th Scientific-Technical Conference Material Problems in Civil Engineering (MATBUD’2015). Procedia Eng..

[27] Chomicz-Kowalska A., Iwański M.M., Mrugała J. (2017). Basic Performance of Fibre Reinforced Asphalt Concrete with Reclaimed Asphalt Pavement Produced in Low Temperatures with Foamed Bitumen. WMCAUS. IOP Conf. Ser. Mater. Sci. Eng..

[28] Hugo Silva M.R.D., Oliveira J.R.M., Peralta J., Zooro S.E. (2010). Optimization of warm mix asphalt using different blends of binders and synthetic paraffin wax contents. Constr. Build. Mater..

[29] Vaitkus A., Čygas D., Laurinavičius A., Perveneckas Z. (2009). Analysis and Evaluation of Possibilities for the Use of Warm Mix Asphalt in Lithuana. Balt. J. Road Bridge Eng..

[30] Chomicz-Kowalska A., Gardziejczyk W., Iwański M.M. (2017). Analysis of IT-CY Stiffness Modulus of Foamed Bitumen Asphalt Concrete Compacted at 95 °C. Modern Building Materials, Structures and Techniques, MBMST 2016. Procedia Eng..

[31] Sanchez-Alonso E., Vega-Zamanillo A., Castro-Fresno D., Del Rio-Prat M. (2011). Evaluation of compactability and mechanical properties of bituminous mixes with warm additives. Constr. Build. Mater..

[32] Iwański M., Mazurek G. (2012). Optimization on the Syntetic Wax Content on Example of Bitumen 35/50. Procedia Eng..

[33] Pszczola M., Jaczewski M., Rys D., Jaskula P., Szydlowski C. (2018). Evaluation of Asphalt Mixture Low-Temperature Performance in Bending Beam Creep Test. Materials.

[34] Iwański M.M., Chomicz-Kowalska A., Maciejewski K. (2020). Resistance to Moisture-Induced Damage of Half-Warm-Mix Asphalt Concrete with Foamed Bitumen. Materials.

[35] Stefańczyk B., Mieczkowski P. (2008). Mieszanki Mineralno-Asfaltowe: Wykonawstwo i Badania. (Bituminous Mixtures: Performance and Research).

[36] Iwański M.M., Chomicz-Kowalska A., Maciejewski K. (2019). Effect of Surface Active Agent (SAA) on 50/70 Bitumen Foaming Characteristics. Materials.

[37] Piłat J., Radziszewski P. (2010). Nawierzchnie Asfaltowe: Podręcznik Akademicki. (Asphalt Pavements Academic handbook).

[38] Luxemburk F. Lime Hydrate as an Additive to Improve the Adhesion of Bitumen to the Aggregates. Proceedings of the II International Conference Durable and Save Road Pavements.

[39] Zou J., Isola M., Roque R., Chun S., Koh C., Lopp G. (2013). Effect of hydrated lime on fracture performance of asphalt mixture. Constr. Build. Mater..

[40] Blazek J., Debor G., Maxa D., Ajib M., Paniagua H. (2000). Effect of hydrated lime addition on properties of asphalt. Pet. Coal.

[41] Jamshidi A., Hamzah M.O., You Z. (2013). Performance of Warm Mix Asphalt containing Sasobit®: State-of-the-art. Constr. Build. Mater..

[42] Iwański M., Mazurek G. (2015). Effect of Fischer-Tropsch synthetic wax additive on the functional properties of bitumen. Polimery.

[43] Butz T., Rahimian I., Hildebrand G. (2001). Modification of road bitumens with the Fischer-Tropsch Paraffin Sasobit. J. Appl. Asph. Bind. Technol..

[44] WT-2 (2014). Technical Guidelines 2: Asphalt Pavements for National Roads.

[45] Judycki J., Jaskuła P., Pszczoła M., Alenowicz J., Dołżycki B., Jaczewski M., Ryś D., Stienss M. (2014). Katalog Typowych Konstrukcji Nawierzchni Podatnych i Półsztywnych (Catalogue of Typical Flexible and Semi-Rigid Pavement Constructions).

[46] EN 1426:2015-08 Bitumen and Bituminous Binders—Determination of Needle Penetration.

[47] EN 1427:2015-08 Bitumen and Bituminous Binders—Determination of Softening Point—Ring and Ball Method.

[48] EN 12593:2015-08 Bitumen and Bituminous Binders—Determination of the Fraass Breaking Point.

[49] EN 13302-2018 Bitumen and Bituminous Binders - Determination of dynamic viscosity of bituminous binder using a rotating spindle apparatus.

[50] Mazurek G. (2012). Optymalizacja składu betonu asfaltowego modyfikowanego woskiem syntetycznym w zakresie odkształceń plastycznych. [Optimization of the asphalt concrete aggregate modified with synthetic wax in the field of plastic deformation]. Ph.D. Thesis.

[51] Mazurek G., Iwański M. (2016). Analysis of selected properties of asphalt concrete with synthetic wax. Bull. Pol. Acad. Sci. Tech. Sci..

[52] (2014). Technical recommendation IBDiM No. RT/2009-03-0012/1. Adhesion Promoters Wetfix BE and Wetfix AP17 for Use in Traffic Engineering.

[53] WETFIX BE (2010). Thermally Stable Liquid Adhesion Promoter for Asphalt Binders.

[54] EN 12594:2014-12 Bitumen and Bituminous Binders—Preparation of Test Samples.

[55] EN 12591:2009 Bitumen and Bituminous Binders—Specifications for Paving Grade.

[56] STATISTICA 13.3 Statsoft. www.statsoft.com.

[57] Piasta Z., Lenarcik A. (1998). Methods of statistical multi-criteria optimization. Optimization Methods for Material Design of Cement-based Composites.

[58] Koronacki J., Mielniczuk J. (2004). Statystyka dla Studentów Kierunków Technicznych i Przyrodniczych. (Statistics for Technical and Natural Sciences Students).

[59] Asphalt Academy (2009). Technical Guideline TG2: Bitumen Stabilised Materials, a Guideline for the Design and Construction of Bitumen Emulsion and Foamed Bitumen Stabilised Materials.

[60] Chomicz-Kowalska A., Maciejewski K. (2015). Multivariate optimization of recycled road base cold mixtures with foamed bitumen. Procedia Eng..

